# Potential Key Markers for Predicting the Prognosis of Gastric Adenocarcinoma Based on the Expression of Ferroptosis-Related lncRNA

**DOI:** 10.1155/2022/1249290

**Published:** 2022-04-29

**Authors:** Yanqun Cai, Susu Wu, Yifan Jia, Xiao Pan, Caiqin Li

**Affiliations:** Taizhou Municipal Hospital, Taizhou 318000, China

## Abstract

**Background:**

Gastric cancer is one of the most common malignant tumors, and it ranks third in global cancer-related mortality. This research was aimed at identifying new targeted treatments for gastric adenocarcinoma by constructing a ferroptosis-related lncRNA prognostic feature model.

**Methods:**

The gene expression profile and clinical data of gastric adenocarcinoma patients were downloaded from TCGA database. FerrDb database was used to determine the expression of iron death-related genes. We used R software to clean the TCAG gastric adenocarcinoma gene expression cohort and screen iron death-related differential genes and lncRNAs. The potential prognostic markers and immune infiltration characteristics were determined by constructing prognostic model and multivariate validation of lncRNA related to ferroptosis prognosis. Finally, the characteristics of immune infiltration were determined by immune correlation analysis.

**Results:**

We identified 26 ferroptosis-related lncRNAs with independent prognostic value. The Kaplan-Meier analysis identified high-risk lncRNAs associated with poor prognosis of STAD. The risk scoring model constructed by AC115619.1, AC005165.1, LINC01614, and AC002451.1 was better than traditional clinicopathological features. The 1-, 3-, and 5-year survival rates of STAD patients were predicted by the nomogram. GSEA reveals the oxidative respiration and tumor-related pathways in different risk groups. Immune analysis found significant differences in the expression of immune checkpoint-related genes TNFSF9, TNFSF4, and PDCD1LG2 between the two groups of patients. Meanwhile, there were significant differences in APC co stimulation, CCR, and checkpoint between the two groups.

**Conclusion:**

Based on the prognostic characteristics of ferroptosis-related lncRNAs, we identified the potential ferroptosis-related lncRNAs and immune infiltration characteristics in gastric adenocarcinoma, which will help provide new targeted treatments for gastric adenocarcinoma.

## 1. Background

Gastric cancer is the fifth most common malignant tumor in the world, second only to lung cancer, breast cancer, colorectal cancer, and prostate cancer [1, 2]. At present, although the incidence of gastric cancer is showing a downward trend, gastric cancer-related mortality is still the third leading cause of cancer-related deaths in the world [3]. As we all know, the main histological type of gastric cancer is adenocarcinoma, and it accounts for more than 95% of all gastric cancers [4]. Risk factors for gastric cancer include Helicobacter pylori infection, age, high salt intake, and a diet low in fruits and vegetables [5, 6]. Although the incidence and mortality of gastric cancer have declined in recent years, and important progress has been made in epidemiology, pathology, pathogenesis, and treatment options, the diagnosis of gastric cancer is often at an advanced stage and still causes high mortality (in 2018, there were 784,000 deaths worldwide) and a huge medical burden [1, 6, 7]. Radiation therapy, chemotherapy, and surgical resection are all currently available therapies for stomach cancer. Unfortunately, the 5-year survival rate for patients with stomach cancer is still quite low. For example, the 5-year overall survival rate of GC patients who receive only treatment is 20% and 30-50% in patients who receive surgery and adjustment therapy, respectively [8, 9]. Therefore, finding new gastric cancer-specific biomarkers is crucial to improve the treatment and prognosis of gastric cancer.

Ferroptosis is a new type of cell death that is different from apoptosis. It mainly involves the accumulation of iron-dependent lipid peroxides (lipid-ROS) and ultimately leads to cell damage [10, 11]. Studies have shown that the imbalance of ferroptosis is related to a variety of pathological changes and diseases, such as ischemia/reperfusion injury (IRI) [12], neurological diseases [13], and cancers [14]. Iron metabolism problems, which raise the risk of cancer and encourage tumor cell proliferation, are associated with this disease. Cancer cells are more reliant on iron than normal cells in order to survive. Cancer cells become addicted to iron, which is a process known as iron addiction [15, 16]. It can be considered that based on the mechanism of ferroptosis in the occurrence of cancer, regulating ferroptosis-related pathways may create new opportunities for cancer treatment strategies [17, 18]. As we all know, 75% of the DNA in the human genome is transcribed into RNA, but only about 2% of the genome encodes proteins, and 98% of the transcripts are noncoding RNA (lncRNA) [19, 20]. Long noncoding RNA (lncRNA) is a kind of RNA molecule, mainly involved in the regulation of gene function [21]. At the same time, lncRNA is also involved in the regulation of various other biological processes, including tumor occurrence, development, and metastasis related processes [22]. Studies have shown that the presence of lncRNA in gastric cancer is involved in the proliferation, migration, invasion, and immune escape of cancer cells, including lncRNA LINC00978, lncRNA ZFAS1, and lncRNA HAGLROS [23–25]. However, there are currently few studies on the molecular characteristics related to ferroptosis to predict the overall survival (OS) of STAD patients. In our research, we obtained differentially expressed genes from the STAD dataset in The Cancer Genome Atlas (TCGA) and constructed a prognostic model of ferroptosis-related lncRNA. We determined the characteristic relationship of ferroptosis-related lncRNA in the prognosis of gastric adenocarcinoma. Finally, we explored the prognostic role of ferroptosis-related lncRNA and immune infiltration in gastric adenocarcinoma. This may provide new insights for the prognosis and treatment of gastric adenocarcinoma.

## 2. Materials and Methods

### 2.1. Manuscript Statement

The paper has been published as a preprint in Research Square [26].

### 2.2. Data Collection and Preprocessing

The Cancer Genome Atlas (TCGA) database is an international public database that aims to research and discover the main oncogenic genome changes of a variety of human tumors through large-scale genome sequencing and comprehensive multidimensional analysis [27]. These publicly available cancer genome data sets will help improve tumor diagnosis methods, treatment standards, and ultimately prevent cancer [27]. We downloaded the gene expression data and clinical data of gastric cancer patients from the TCGA database.

### 2.3. Identification and Coexpression Analysis of Ferroptosis -Related Genes and lncRNA

We downloaded ferroptosis-related genes from the FerrDb database, which is an experimentally verified database of ferroptosis regulators and markers and the association between ferroptosis and disease [28]. We use the limma package to perform differential analysis on ferroptosis-related genes, and the screening criterion is ∣ log 2 FC | ≥1, *P* value < 0.05 [29]. Pearson correlation was used to evaluate the coexpression relationship between ferroptosis-related lncRNA and gastric adenocarcinoma. Determine the correlation coefficient ∣ *R* 2 | >0.3 as *P* value < 0.001. The significant differential expression of ferroptosis-related lncRNA is set to FDR < 0.05 and ∣ log 2 FC | ≥1.

### 2.4. GO and KEGG Enrichment Analysis

First, we explored the function of upregulated and downregulated differential genes related to ferroptosis. We use Gene Ontology (GO) to evaluate the biological pathways of differential genes related to ferroptosis. Based on the Kyoto Encyclopedia of Genes and Genomes (KEGG) data, the R software ggplot2 package is used to plot biological processes (BP), molecular functions (MF), and cell components (CC) regulated by differently expressed ferroptosis-related differential genes.

### 2.5. Screening of Prognostic-Related lncRNAs and Construction of Prognostic Models

We use the R software limma package to merge the lncRNA expression level and the survival data of gastric adenocarcinoma patient samples with the correlation coefficient filter standard corfilter < −0.4, *P* value Filter < -0.001 as the setting standards [29]. Use the survival package of the R software, *P* Filter = 0.05 to visualize the HR value of lncRNA, and use the ggplot2 package to draw prognostic-related lncRNA forest maps. We identified the prognostic-related lncRNAs, using _P_ value < 0.05 as the screening criteria. We use the stepped AIC algorithm to select the prognostic model. The model formula is (coefficient lncRNA1 × lncRNA1 expression) + (coefficient lncRNA2 × lncRNA2 expression) + ⋯+(coefficient lncRNAn × expression lncRNA). According to the formula and sample expression, we obtain the risk score of the sample, and divide the patients into high and low risk groups according to the median value of the risk score.

### 2.6. Multivariate Validation of Prognostic Model

The Kaplan-Meier curve is a common method for dealing with various survival time analyses; survival analysis is used to calculate and visualize survival probability, particularly when some subjects cannot continue the study [30, 31]. We used the Kaplan-Meier survival analysis to evaluate the survival probability of STAD patients based on ferroptosis-related lncRNAs characteristics. We draw survival curves by using the R software survival package, survminer package, and ggsurvplot. Based on unique COX analysis to determine the correlation between clinical factors and patient prognosis, the multivariate COX analysis predicts independent prognostic factors; setting *P* value < 0.05 is significant. We use operating characteristic curve (ROC) and decision curve analysis (DCA) to assess the difference between the prognostic characteristics of STAD and the sensitivity and specificity of clinical pathology [32, 33]. We analyze the relationship between ferroptosis-related lncRNAs and differential gene coexpression by constructing a coexpression network [34].

### 2.7. Prognosis Nomogram and GSEA Enrichment Analysis

Based on the TCGA-STAD database, set the statistical significance to *P* value < 0.05 and false discovery rate (FDR) *q* < 0.25, we constructed a nomogram with prognostic characteristics to predict the 1-, 3-, and 5-year survival of STAD patients rate [35]. We use gene set enrichment analysis (GSEA) to analyze the lncRNA characteristics of ferroptosis-related lncRNA in GO, KEGG, and HALLMARK [36].

### 2.8. Immune Cell Correlation Analysis

Based on a variety of immune analysis algorithms, including TIMER, CIBERSORT, CIBERSORT-ABS, QUANTISEQ, MCPcounter, XCELL, and EPIC algorithms for immune analysis comparison [37, 38], to evaluate the cellular immune characteristics of the iron-death phase lncRNA between the high-risk and low-risk groups, we show the differences in immune response under different algorithms by drawing immune correlation heat maps. In addition, ssGSEA is used to quantify the tumor-infiltrating immune cell subsets between the two groups and assess their immune function.

## 3. Result

### 3.1. Data Collection and Identification of Ferroptosis-Related Genes and lncRNA and Coexpression Analysis

We downloaded the gene expression data and clinical data of 407 gastric cancer patients (53 normal cases and 354 tumor cases) from the TCGA database. The collected clinicopathological data of patients with gastric adenocarcinoma include gender, age, stage, grade, TMN, survival status, and survival time. The clinical characteristics of the patients are shown in Table 1. Based on the FerrDb database, we downloaded 259 ferroptosis-related genes (driver: 108; inhibitor: 69; marker: 111). We identified 1849 lncRNAs associated with ferroptosis for coexpression analysis.

### 3.2. Analysis of GO and KEGG Enrichment of DEG Related to Ferroptosis

**Figure 1:**
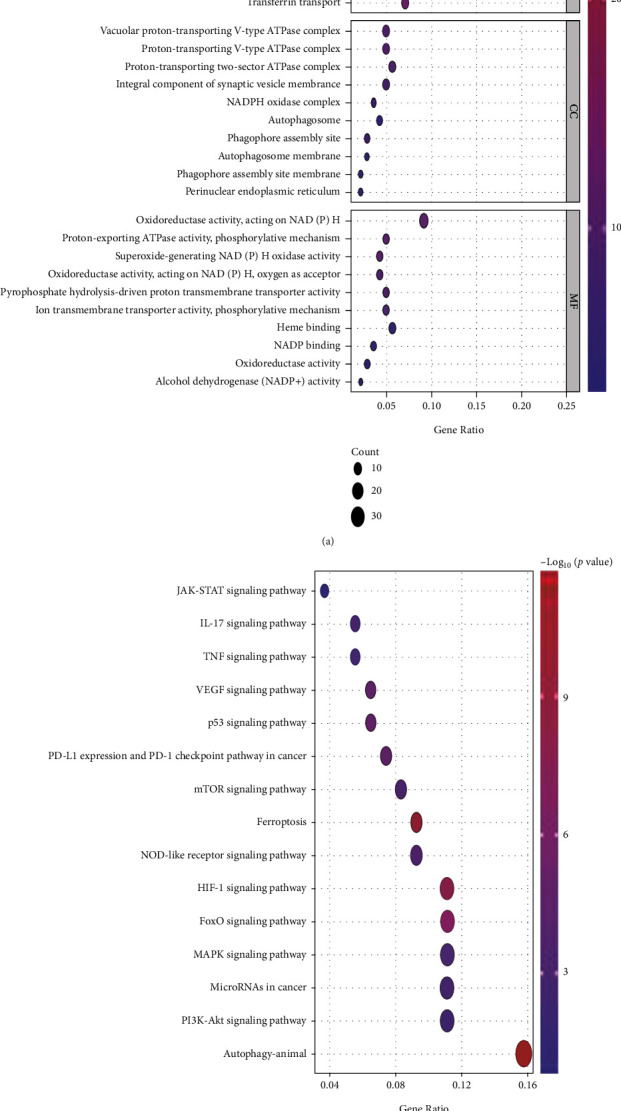
GO and KEGG analyses of differentially expressed genes related to ferroptosis. (a) GO analysis results. The color of the dots represents the adjusted *P* value: red, low; blue, high. The size of the dots represents the number of ferroptosis-related genes. (b) KEGG pathway enrichment analyses. The color of the dots represents the adjusted *P* value, and the size of the dots represents the number of ferroptosis-related genes in the pathway. GO: Gene Ontology; KEGG: Kyoto Encyclopedia of Genes and Genomes; BP: biological process; CC: cellular component; MF: molecular function.

We identified 137 different genes related to ferroptosis (61 downregulated, 76 upregulated). GO enrichment shows that biological processes (BP) are mainly involved in cellular response to chemical stress, cell response to oxidative stress, regulation of autophagy, and iron ion transport. Molecular function (MF) mainly regulates the NADPH oxidase activity that produces superoxide, ion transmembrane transport protein activity, phosphorylation mechanism, and NADP binding. Cellular components (CC) are mainly in vacuolar proton transport type V ATPase complex, synaptic vesicle membrane component, NADPH oxidase complex, and autophagosome. Based on KEGG analysis, overexpressed genes are mainly involved in autophagy-animals, ferroptosis, HIF-1 signaling pathway, FoxO signaling pathway, VEGF signaling pathway, PD-L1 expression and PD-1 checkpoint pathway in cancer, mTOR signaling pathway, MAPK signaling pathway, PI3K-Akt signaling pathway, TNF signaling pathway, and JAK-STAT signaling pathway (Figures 1(a) and 1(b)).

### 3.3. Screening and Model Construction of Prognostic-Related lncRNAs

**Figure 2:**
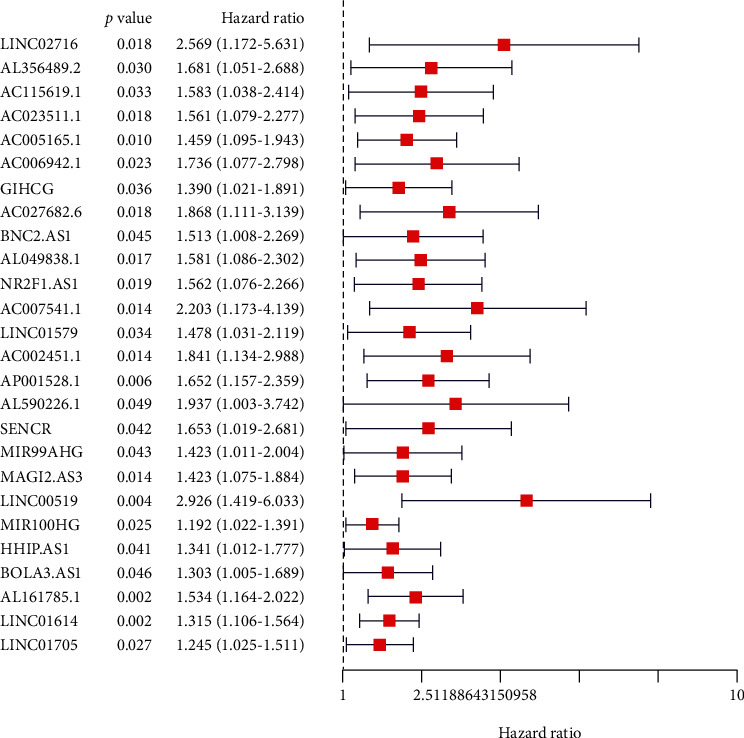
26 independent prognostic predictor lncRNA signatures with different expressions of STAD.

We identified 26 different expressed lncRNA signatures as independent prognostic predictors of STAD, by screening prognostic-related lncRNAs. These lncRNAs include LINC02716, AL356489.2, AC115619.1, AC023511.1, AC005165.1, AC006942.1, GHICG, AC027682.6, BNC2.AS1, AL049838.1, NR2F1.AS1, AC007541.1, LINC01579, AC002451.1, AP001528.1, AL590226.1, SENCR, MIR99AHG, MAGI2.AS3, LINC00519, MIR100HG, HHIP.AS1, BOLA3.AS1, AL161785.1, LINC01614, and LINC01705. We calculated the lncRNA signature risk score and constructed a prognostic feature model. Finally, we choose AC115619.1, AC005165.1, LINC01614, and AC002451.1 as the construction risk scoring genes, risk score = (0.5518∗AC115619.1.EXP) + (0.3165∗AC005165.1.EXP) + (0.3277∗LINC01614.EXP) + (0.5196∗AC002451.1.EXP). The forest plot shows that these lncRNAs are significant in predicting prognosis (Figure 2).

We performed univariate Cox regression analyses and identified 26 ferroptosis-related lncRNAs associated with STAD patient prognosis. Red color represents high *P* values. 26 independent prognostic predictor lncRNA signatures with different expressions of STAD.

### 3.4. Multivariate Validation of Prognostic Model

**Figure 3:**
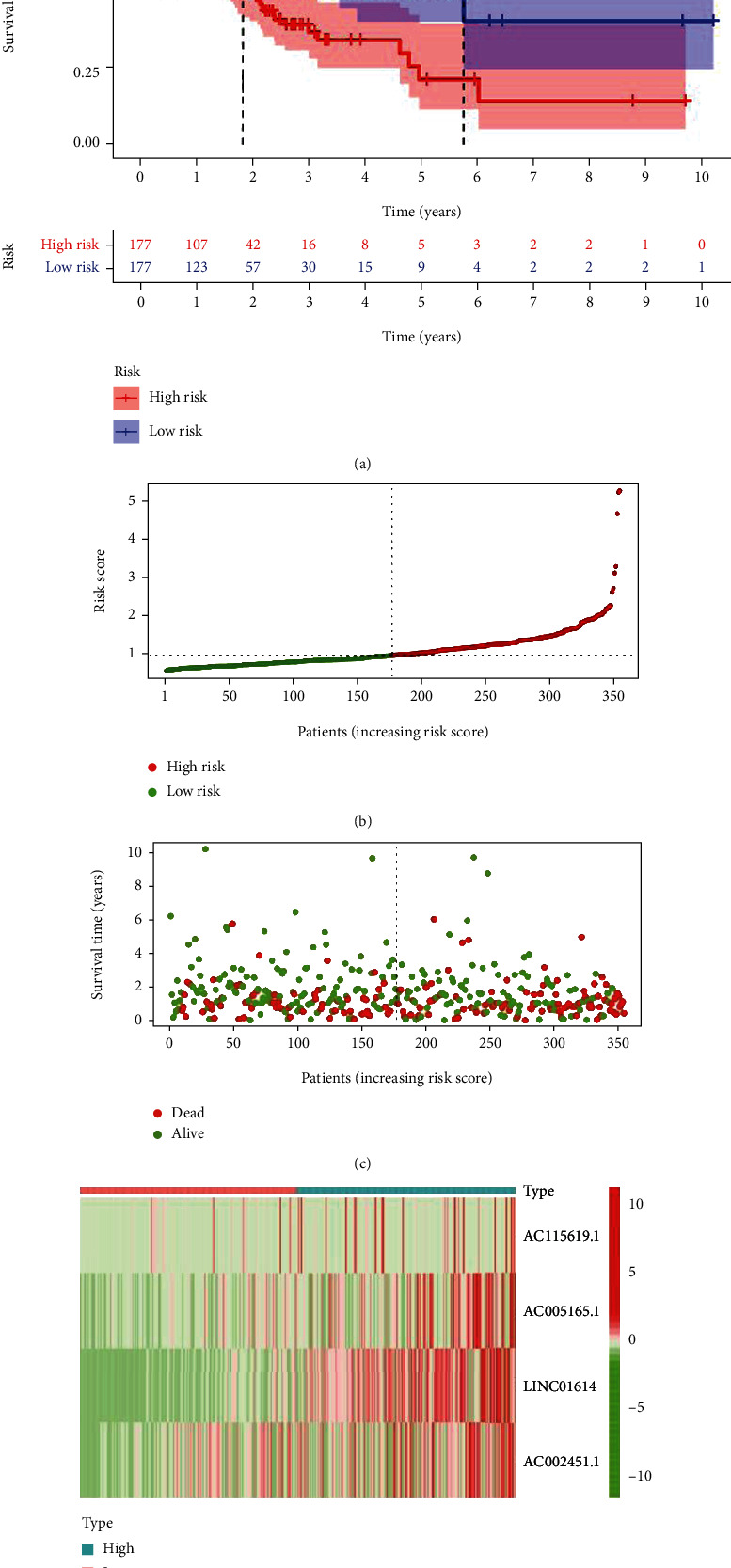
Feature of ferroptosis-related lncRNAs. (a) The Kaplan-Meier curve showed that patients with high risk displayed a shorter overall survival than those with low risk. (b) The median value and distribution of the risk scores. (c) The distribution of OS status. (d) Heat map of four lncRNAs.

Based on the Kaplan-Meier analysis, the prognostic model showed that the high-risk group lncRNAs had a worse survival rate than the low-risk group lncRNAs (*P* < 0.05) (Figure 3(a)). Through the patient's risk curve and scatter plot, we found that the patient's risk score is inversely proportional to the survival rate of patients with gastric adenocarcinoma (Figures 3(b) and 3(c)). Convincingly, our risk heat map shows that there are four lncRNAs that are highly expressed in the high-risk group and are significantly positively correlated with our risk model, including AC115619.1, AC005165.1, LINC01614, and AC002451.1 (Figure 3(d)).

### 3.5. The Analysis of Independent Prognostic

**Figure 4:**
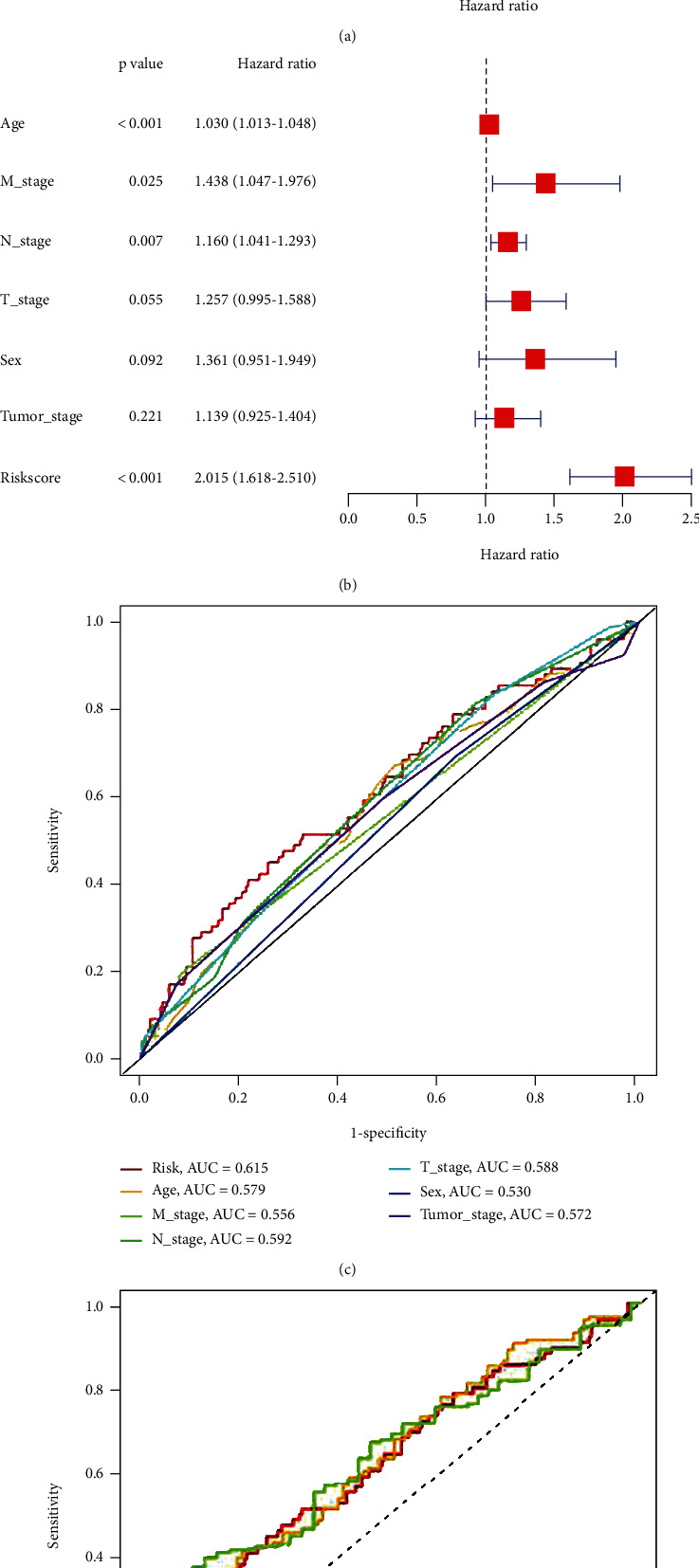
Univariate and multivariate COX analyses and prognostic characteristics of ferroptosis-related lncRNAs. (a) Univariate Cox analysis of ferroptosis-related lncRNA expression, (b) multivariate Cox analysis of ferroptosis-related lncRNA expression, (c) AUC value of lncRNA risk characteristics, and (d) AUC value of lncRNA risk characteristics were used to predict the 1-, 3-, and 5-year survival rates of STADs.

In our prognosis model, univariate and multivariate Cox analyses revealed lncRNA characteristics (HR: 2.015, 95CI: 1.618-2.510), patient age (HR: 1.030, 95CI: 1.013-1.048), and tumor M stage (HR: 1.438, 95CI: 1.618-2.510). 1.047-1.976) and tumor N staging (HR: 1.160, 95CI: 1.041-1.293) were independent prognostic factors for OS in patients with STAD (Figures 4(a) and 4(b)). The cliROC curve shows that the AUC of the risk feature lncRNA is 0.615, indicating that it is better than other traditional clinicopathological features in predicting the prognosis of STAD. At the same time, the survival rate ROC curve showed that the 1-, 2-, and 3-year survival rates of lncRNAs in STAD patients were 0.615, 0.631, and 0.638, respectively (Figures 4(c) and 4(d)).

**Figure 5:**
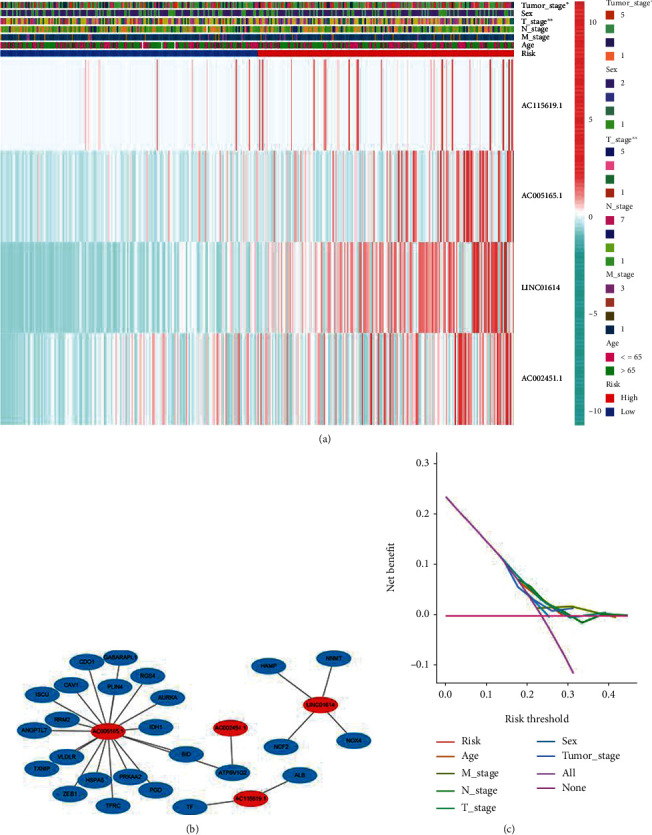
The analysis of the correlation between the prognostic characteristics of ferroptosis-related lncRNA and clinicopathology. (a) Heat map of the prognostic characteristics and clinicopathological correlation of ferroptosis-related lncRNAs. (b) Coexpression network: the red nodes represent ferroptosis-related lncRNAs, and the green nodes represent ferroptosis-related genes. (c) DCA for different risk factors.

At the same time, we analyzed the correlation between the prognostic characteristics of lncRNAs associated with ferroptosis and the clinicopathological manifestations. The correlation heat map showed that AC115619.1, AC005165.1, LINC01614, and AC002451.1 were significantly associated with a higher-risk group of STAD patients, as shown in Figure 5(a). The coexpression relationship between lncRNA and mRNA was shown in Figure 5(b). Interestingly, the DCA curve of our risk model shows that the performance of lncRNAs in predicting the prognosis of STAD compared with other traditional clinicopathological features still needs more experimental studies, as shown in Figure 5(c).

### 3.6. The Nomogram of Prognosis-Related

**Figure 6:**
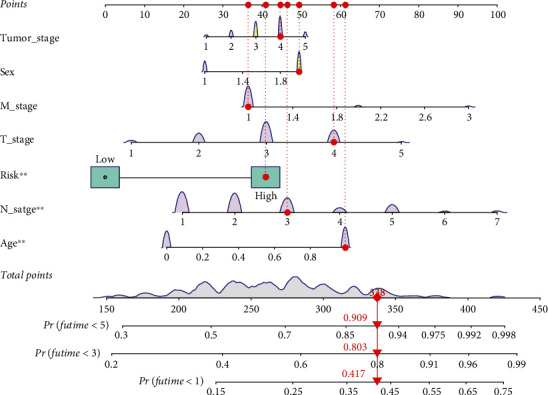
The nomogram of traditional clinicopathological factors and ferroptosis-related prognostic lncRNAs.

We predicted 1-, 3-, and 5-year survival rates in patients with STAD by combining clinicopathological features of STAD patients with prognostic features of ferroptosis-related lncRNAs. We score each traditional clinical trait and prognosis model individually. Through comprehensive scoring, we can predict the survival probability of patients, which is helpful to clinically guide the management and treatment of STAD patients (Figure 6).

Through comprehensive scoring, the nomogram can predict the survival probability of STAD patients at 1, 3, and 5 years to be 0.417, 0.803, and 0.909, respectively.

### 3.7. GSEA Enrichment Analysis

**Figure 7:**
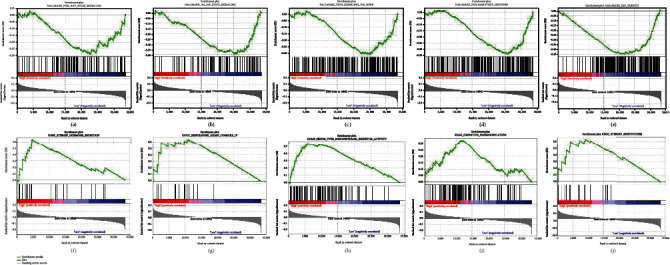
Gene enrichment analysis of ferroptosis-related lncRNA based on TCGA-STAD.

Our research revealed that most of the new ferroptosis-related lncRNA prognostic characteristics on gene set enrichment analysis (GSEA) regulate oxidative respiration and tumor-related pathways, such as PI3K-AKT-MTOR signal, IL6-JAK-STAT3 signal, and NFKB TNFA signal, inflammatory response, E2F target, steroid hormone secretion, respiratory chain complex IV, serine endopeptidase inhibitor activity, oxidative phosphorylation, and steroid biosynthesis (Figure 7).

### 3.8. The Analysis of Immune Correlation

**Figure 8:**
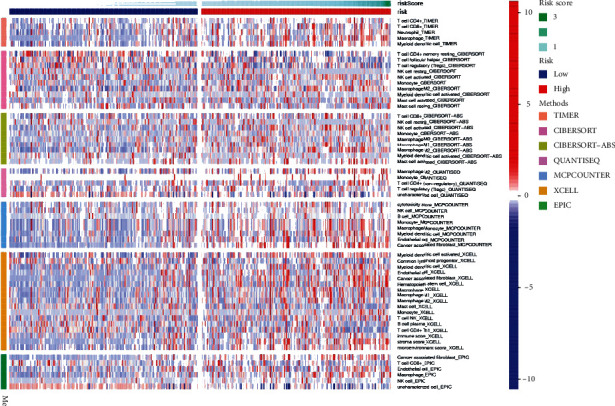
Heat maps of the high- and low-risk group immune responses based on Timer, Cibersort, Cibersort-abs, Quantiseq, MCPCounter, Xcell, and EPIC algorithms. Red represents high expression of immune cells, and blue represents low expression of immune cells.

We have used a variety of immune analysis algorithms, including algorithms for immune analysis and comparison, and drawn the immune response heat map as shown in Figure 8.

**Figure 9:**
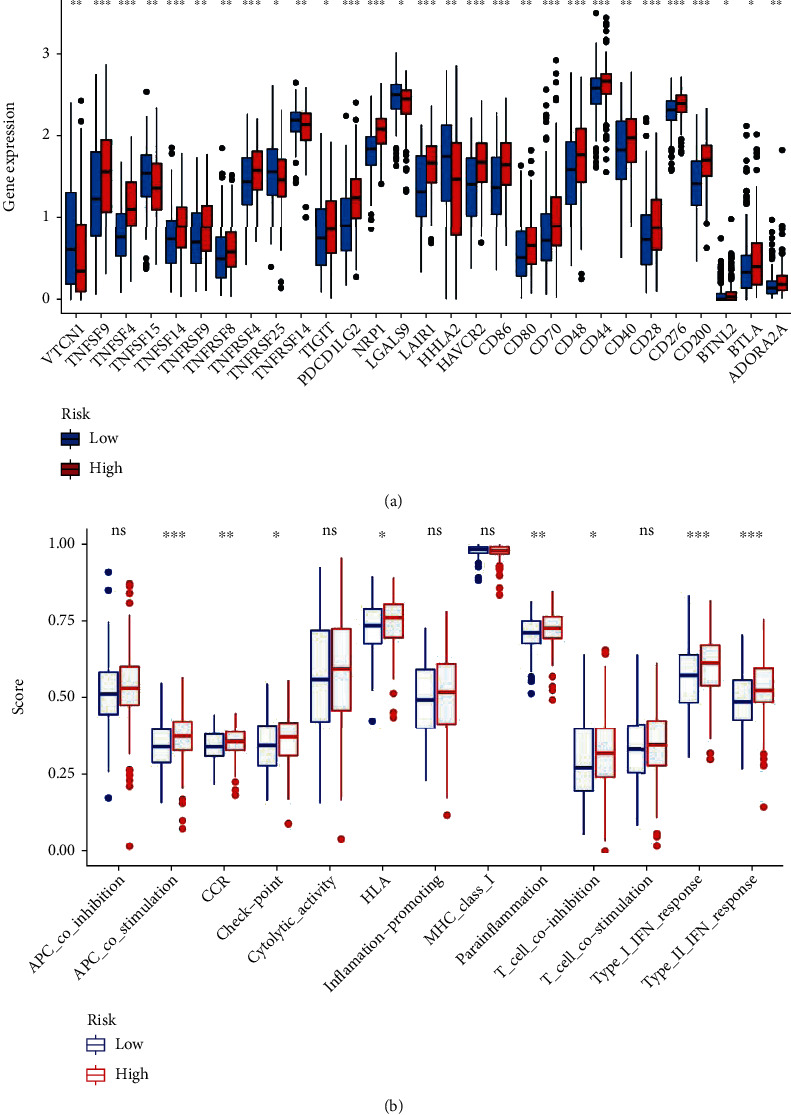
The immune analysis between high and low STAD risk groups. (a) Expression at immune checkpoints in the high and low STAD risk groups. (b) Differential associations between immune cell subsets and related functions.

We use a variety of immune analysis algorithms, including TIMER, CIBERSORT, CIBERSORT-ABS, QUANTISEQ, MCPcounter, XCELL, and EPIC algorithm for immune analysis comparison, and draw the immune reaction heat map as shown in Figure 8. Based on the importance of checkpoint inhibitors in immunotherapy for patients with STAD, we analyzed differences in immune checkpoint expression between high- and low-risk groups. We found significant differences in the expression of immune checkpoint-related genes TNFSF9, TNFSF4, PDCD1LG2, NRP1, LAIR1, HAVCR2, CD86, CD48, CD200, etc., between the two groups of patients (Figure 9(a)). At the same time, we analyzed the correlation between the immune cell subsets of ssGSEA and related functions based on TCGA-STAD data, showing that APC-costimulation (antigen-presenting cell costimulation), CCR, check-point, HLA, parainflammation, T cell coinhibition, type II INF response, and type II INF response are significant between the high-risk and low-risk group difference (Figure 9(b)).

## 4. Discussion

As we all know, ferroptosis is a cell death process that is different from apoptosis, pyrolysis, and various forms of cell necrosis in morphology, biochemistry, and genetics [39]. Studies have shown that iron overload is related to the development of cancer, which leads to DNA damage and promotes tumorigenesis through prooxidation [40, 41]. Interestingly, there are also studies that believe that ferroptosis can eliminate the adaptive characteristics of malignant cells and remove cells that cannot obtain key nutritional factors and are infected or destroyed by environmental changes. Based on the key role of ferroptosis in inhibiting tumorigenesis, it can be considered that it may be a new direction of tumor treatment. In our study, new lncRNA signatures for ferroptosis-related prognosis were identified based on TCGA STAD patient data, including LINC01614, AC005165.1, AC002451.1, and AC115619.1. Then, we evaluated the role of immune infiltrating cells and immune checkpoint inhibitors in the tumor microenvironment in the prognosis of STAD. In conclusion, our research provides new insights for STAD-related ferroptosis-related lncRNA as potential biomarkers and therapeutic targets.

The enrichment analysis of 137 ferroptosis-related differential genes shows that KEGG is mainly enriched in autophagy-animals, ferroptosis, HIF-1 signaling pathway, FoxO signaling pathway, VEGF signaling pathway, PD-L1 expression and PD-1 inspection point pathway in cancer, MAPK signaling pathway, PI3K-Akt signaling pathway, and JAK-STAT signaling pathway. Recent studies have shown that the protective effect of FG-4592 (HIF prolyl hydroxylase inhibitor) pretreatment is mainly through Akt/GSK-3 *β*-mediated stabilization of HIF-1*α* and activation of the Nrf2 signaling pathway to reduce folic acid (FA) induction of ferroptosis in the early stages of kidney injury [42]. Meanwhile, Tyro3 promotes the development of the original tumor microenvironment by inhibiting ferroptosis of tumor cells induced by anti-PD-1/PD-L1 and reducing the M1/M2 macrophage ratio, thus leading to resistance to PD-1/PD-L1 therapy [43]. Studies have shown that oncogenic activation of the PI3K-Akt-mTORC1 pathway leads to downstream SREBP1 (sterol regulatory element-binding protein 1)/SCD1 (stearyl coenzyme A desaturase-1)-mediated lipogenesis that inhibits ferroptosis in cancer cells [44]. In conclusion, this study identified 26 ferroptosis-related lncRNA in gastric adenocarcinoma samples as independent prognostic predictors of STAD. Studies have shown that lnc-GIHCG overexpression increases the proliferation and migration of gastric cancer cells by upregulating TLE1 expression through the adsorption of miR-1281 [45]. Liu C. et al. experimentally showed that knockdown of lncRNA BNC2-AS1 significantly inhibited the proliferation and migration of gastric cancer cells [46]. Studies have shown that lncRNA MIR99AHG induces EMT and inhibits apoptosis through miR577/FOXP1 axis to promote gastric cancer progression [47]. Our study determined that LINC01614, AC005165.1, AC002451.1, and AC115619.1 were significantly related to the high-risk group of gastric adenocarcinoma. Convincingly, some studies also believe that LINC01614 is of great significance in the diagnosis and prognosis of gastric cancer [48]. Chen Y. et al. verified that LINC01614 is highly expressed in GC cell lines and low in normal cells through semiquantitative PCR experiments and concluded that LINC01614 has carcinogenic effects in promoting the growth and migration of GC cells [48]. At present, the mechanism of LINC01614 in GC is not yet clear. Interestingly, there are some studies on the mechanism of action of LINC01614 in other tumors. Liu et al. experimentally proved that LINC01614 mediates inhibition of miR-217 and promotes FOXP1, which ultimately stimulates the development of lung adenocarcinoma (LUAD) [49]. Wang et al. predicted LINC01614 as a potential biomarker for prognosis of breast cancer (BRCA) through Cox analysis [50]. Based on the coexpression analysis of the prognostic-related ferroptosis lncRNA, we found that the expression of LINC01614 is correlated with NCF2, NOX4, HAMP, and NNMT. Among them, NOX4 plays an important role in the occurrence of cancer. Many studies have shown that NOX4 and its derivatives ROS are closely related to tumorigenesis or carcinogenesis, [51] cancer cell proliferation, [52, 53] tumor metastasis [54, 55], invasion [56, 57], DNA damage [58], and anticancer cell apoptosis [59]. Importantly, studies have shown that NOX4 plays an important role in the growth and apoptosis of gastric cancer cells by producing ROS and activating GLI1 signaling [60]. However, our study found that LINC01614 may play an important role in gastric adenocarcinoma in regulating the ferroptosis process. These findings may provide new directions for the treatment and prognosis of gastric cancer in the future.

In our research, GSEA is enriched in PI3K-AKT-MTOR signal, IL6-JAK-STAT3 signal, TNFA signal through NFKB, inflammatory response, E2F target, oxidative phosphorylation, steroid biosynthesis, and other signal pathways. Previous studies have shown that Forkhead box D1 antisense RNA 1 (FOXD1-AS1) promotes Forkhead box D1 (FOXD1) translation through PIK3CA/PI3K/AKT/mTOR signaling, thereby aggravating gastric cancer progression and chemotherapy resistance [61]. Similarly, cancer-associated fibroblasts (CAF) in the tumor microenvironment promote the progression of gastric cancer through IL-6/JAK2/STAT3 signaling and achieve a targeted therapeutic effect on gastric cancer through the action of IL-6 on stromal fibroblasts [62].

As a new form of cell death, ferroptosis may become a new method of tumor treatment in the future. Based on the interaction of lncRNA with protein, RNA, DNA, or a combination of these to regulate its function in diseases, the key carcinogenic mechanism of lncRNA in human cancer still needs more research [63, 64]. Therefore, this study is based on the analysis of the correlation between ferroptosis and lncRNA and explores the ferroptosis-related lncRNA markers that can be used to predict the prognosis of STAD. This may provide a new direction for the treatment of tumors. Nevertheless, our research still has certain limitations. Since our research results have not been verified by clinical samples, the reliability of the research results cannot be guaranteed. However, our research still has certain guiding significance.

## 5. Conclusion

In conclusion, this study confirmed that the signature of 4 ferroptosis-related lncRNAs might be applied for predicting the prognosis of STAD. In the personalized treatment of STAD patients, the assessment of the degree of hypoxia is strongly recommended to benefit specific patient groups. However, this prognostic 11-lncRNA signature should be validated by experimental studies.

## Data Availability

Publicly available datasets were analyzed in this study. The raw data supporting the conclusions of this article will be made available in online https://portal.gdc.cancer.gov/.
